# Successful Prediction of Human Fetal Exposure to P-Glycoprotein Substrate Drugs Using the Proteomics-Informed Relative Expression Factor Approach and PBPK Modeling and Simulation[Fn fn4]

**DOI:** 10.1124/dmd.121.000538

**Published:** 2021-10

**Authors:** Olena Anoshchenko, Flavia Storelli, Jashvant D. Unadkat

**Affiliations:** Department of Pharmaceutics, University of Washington, Seattle, Washington

## Abstract

Many women take drugs during their pregnancy to treat a variety of clinical conditions. To optimize drug efficacy and reduce fetal toxicity, it is important to determine or predict fetal drug exposure throughout pregnancy. Previously, we developed and verified a maternal-fetal physiologically based pharmacokinetic (m-f PBPK) model to predict fetal K_p,uu_ (unbound fetal plasma AUC/unbound maternal plasma AUC) of drugs that passively cross the placenta. Here, we used in vitro transport studies in Transwell, in combination with our m-f PBPK model, to predict fetal K_p,uu_ of drugs that are effluxed by placental P-glycoprotein (P-gp)—namely, dexamethasone, betamethasone, darunavir, and lopinavir. Using Transwell, we determined the efflux ratio of these drugs in hMDR1-MDCK^cP-gpKO^ cells, in which human P-gp was overexpressed and the endogenous P-gp was knocked out. Then, using the proteomics-informed efflux ratio–relative expressive factor approach, we predicted the fetal K_p,uu_ of these drugs at term. Finally, to verify our predictions, we compared them with the observed in vivo fetal K_p,uu_ at term. The latter was estimated using our m-f PBPK model and published fetal [umbilical vein (UV)]/maternal plasma drug concentrations obtained at term (UV/maternal plasma). Fetal K_p,uu_ predictions for dexamethasone (0.63), betamethasone (0.59), darunavir (0.17), and lopinavir (0.08) were successful, as they fell within the 90% confidence interval of the corresponding in vivo fetal K_p,uu_ (0.30–0.66, 0.29–0.71, 0.11–0.22, 0.04–0.19, respectively). This is the first demonstration of successful prediction of fetal K_p,uu_ of P-gp drug substrates from in vitro studies.

**SIGNIFICANCE STATEMENT:**

For the first time, using in vitro studies in cells, this study successfully predicted human fetal K_p,uu_ of P-gp substrate drugs. This success confirms that the m-f PBPK model, combined with the ER-REF approach, can successfully predict fetal drug exposure to P-gp substrates. This success provides increased confidence in the use of the ER-REF approach, combined with the m-f PBPK model, to predict fetal K_p,uu_ of drugs (transported by P-gp or other transporters), both at term and at earlier gestational ages.

## Introduction

More than half of all pregnant women take drugs (medication) throughout pregnancy, and about 25% take drugs in the first trimester (Scaffidi et al., 2017). Drugs are administered either to treat the mother for various clinical conditions (e.g., depression, epilepsy, gestational diabetes) or to treat her fetus (e.g., to prevent poor lung development in case of preterm delivery or to prevent vertical transmission of HIV) (Sheffield et al., 2014). Despite the high frequency of drug use in pregnancy, little is known about the drug benefits and risks for the fetus, which are related to fetal drug exposure after maternal drug administration. Fetal drug exposure [defined as an area under drug plasma concentration-time profile (AUC)] is determined by maternal drug exposure, placental transport/metabolism, and fetal drug elimination (Zhang et al., 2017). The extent of fetal drug exposure can be evaluated by K_p,uu_, the ratio of fetal to maternal unbound plasma AUCs after single- or multiple-dose drug administration or the corresponding average steady-state plasma concentrations (C_ss_) after multiple-dose administration (eq. 1), where f_u,f_ and f_u,m_ are the fractions of unbound drug in fetal or maternal plasma, respectively).

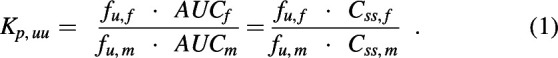


In the absence of placental transport (and fetoplacental metabolism), fetal K_p,uu_ is unity (i.e., drugs passively diffuse across the placenta from the mother to the fetus, yielding equal maternal and fetal unbound plasma AUCs). When placental drug efflux by transporters abundant in the human placenta [e.g., by P-glycoprotein (P-gp) (Mathias et al., 2005; Joshi et al., 2016; Anoshchenko et al., 2020)] is present, K_p,uu_ will be less than unity. Such placental drug efflux can modulate fetal exposure to drugs and, therefore, compromise efficacy (if the fetus is the therapeutic target) or reduce potential fetal toxicity.

To determine fetal K_p,uu_ of a drug at any gestational age, measurement of fetal (and maternal) drug plasma concentrations is necessary. However, except at term, for ethical and logistical reasons, it is impossible to measure fetal (e.g., umbilical vein) drug concentrations. Various in vitro systems have attempted to mimic the syncytiotrophoblast (SYT) placental barrier that could aid in K_p,uu_ estimation (Arumugasaamy et al., 2020), but most of them fail to recapitulate the complexity of SYT layer in vivo (e.g., BeWo, JAR, Jeg-3 cell monolayers), are laborious (perfused human placenta), or are at very early stages of development (microphysiological systems). Because of the limitations of the aforementioned systems and the lack of clinical data at earlier gestational ages, an alternative is to predict, as opposed to measure, fetal K_p,uu_. Such predictions can be made and verified at term using physiologically based pharmacokinetic (PBPK) modeling and simulation (M&S).

**Fig. 1.**
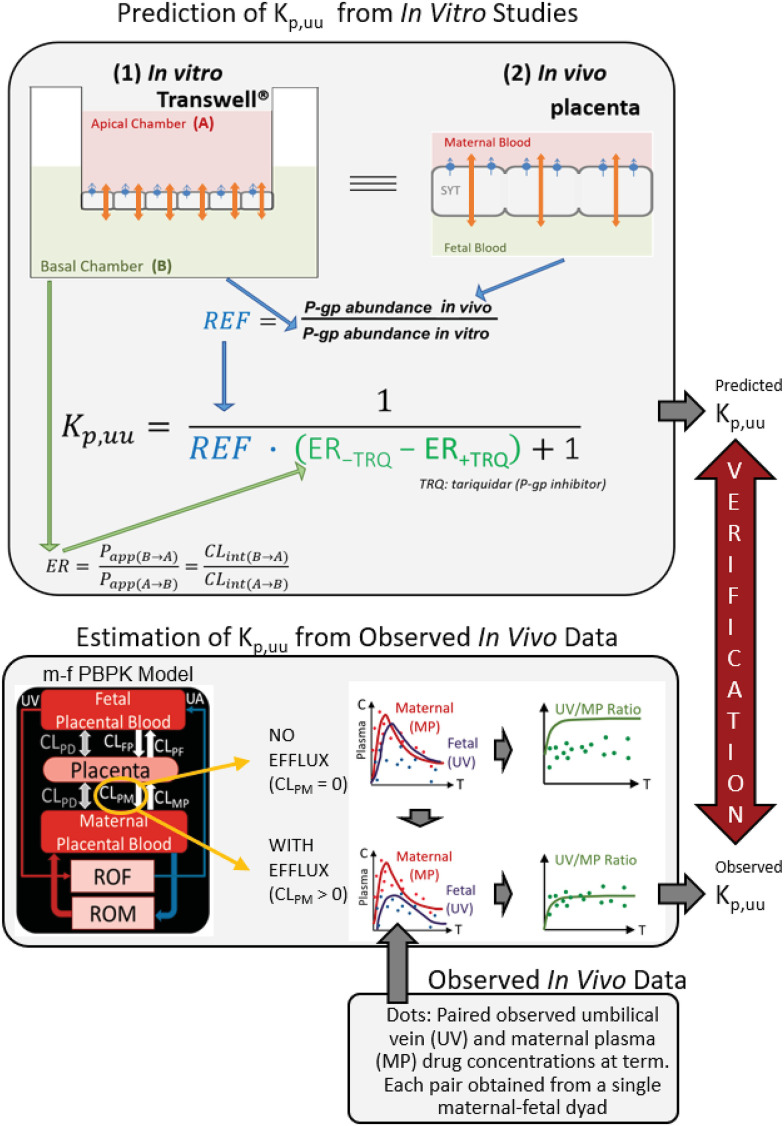
Workflow for the prediction of in vivo fetal K_p,uu_ using the ER-REF approach and subsequent verification of the predicted K_p,uu_ by comparison with the observed in vivo K_p,uu_ estimated by m-f PBPK modeling and simulation. Top panel: efflux transporter-overexpressing cell monolayer (e.g., hMDR1-MDCK^cP-gp KO^) in the in vitro Transwell system (1) mimics the placental SYT layer in vivo (2); that is, the apical and basal chambers in the in vitro system, respectively, mimic the in vivo maternal and fetal blood compartments, allowing the use of the ER-REF approach to predict the in vivo fetal K_p,uu_. For verification, this predicted K_p,uu_ was compared with the observed in vivo K_p,uu_ estimated by m-f PBPK modeling and simulation as depicted in the bottom panel. Orange arrows indicate bidirectional intrinsic passive diffusion clearance. Blue circles and blue arrows respectively represent apically localized efflux transporters and the direction of drug efflux/intrinsic placental-maternal clearance (CL_PM_, specified as CL_int,P-gp,placenta_ in the text). ER-REF is efflux ratio–relative expression factor approach. P_app(B→A)_ and P_app(A→B)_ are apparent permeabilities, and CL_int(B→A)_ and CL_int(A→B)_ are apparent intrinsic clearances of a drug in the indicated directions. Bottom panel: estimation of K_p,uu_ from the observed in vivo data with and without intrinsic active placental-maternal efflux clearance (CL_PM_) incorporated into the model. For drugs that are effluxed by placental P-gp (i.e., CL_PM_ > 0), CL_PM_ was adjusted until the m-f PBPK model–predicted UV/MP values best described the observed UV/MP values (dots). Then, based on eq. 1, the in vivo K_p,uu_ was estimated. CL_PD_, intrinsic passive diffusion clearance; CL_FP_, intrinsic active fetal-placental clearance; CL_PF_, intrinsic placental-fetal clearance; CL_MP_, maternal-placental clearance [assume 0 for drugs transported only by placental-maternal efflux transporters (CL_PM_)]; ROF, rest of the fetal compartment; ROM, rest of the maternal compartments; UA, umbilical artery.

We have previously developed and verified a maternal-fetal physiologically based pharmacokinetic (m-f PBPK) model capable of predicting maternal-fetal exposure to drugs that are metabolized by various cytochrome P450 enzymes (Ke et al., 2012, 2014) and cross the placenta by passive diffusion (Zhang et al., 2017; Zhang and Unadkat, 2017). However, many drugs administered to pregnant women are substrates of efflux transporters that are highly expressed in the placenta, such as P-glycoprotein (P-gp) and breast cancer resistance protein (BCRP) (Mathias et al., 2005; Anoshchenko et al., 2020). Both serve to reduce fetal exposure to drugs such as corticosteroids (Petersen et al., 1980; Tsuei et al., 1980), HIV protease inhibitors (Fauchet et al., 2015; Colbers et al., 2016), or anticancer drugs (e.g., imatinib) (Russell et al., 2007). Therefore, to make our m-f PBPK model comprehensive, we combined it with the efflux ratio–relative expression factor approach (ER-REF) to predict fetal K_p,uu_ of drugs that are actively transported by the placenta. The ER-REF approach to predict K_p,uu_ has been described previously to predict brain distribution of transporter substrates in humans and preclinical species (Uchida et al., 2011, 2014; Trapa et al., 2019; Storelli et al., 2021). It relies on measurement of 1) transport clearance of the drugs [i.e., via the efflux ratio (ER)] in transporter-overexpressing cell lines (e.g., Transwell) and 2) transporter abundance in both in vivo tissue (the placenta) and transporter-overexpressing cell lines using quantitative targeted proteomics to obtain REF (see Fig. 1 for workflow).

Using this ER-REF, combined with our m-f PBPK model, we predicted the fetal K_p,uu_, of four model P-gp substrate drugs—namely, two antenatal corticosteroids (ACS), dexamethasone (DEX) and betamethasone (BET), and two HIV protease inhibitors (PIs), darunavir (DRV) and lopinavir (LPV). Then, to verify our K_p,uu_ predictions, we compared these predictions with the corresponding estimated in vivo fetal K_p,uu_ of these drugs. The latter was estimated from m-f PBPK modeling of the observed maternal and fetal (umbilical vein) plasma concentrations of these drugs, obtained at term (or close to term), in a number of maternal-fetal dyads (Fig. 1).

## Materials and Methods

### Chemicals and Reagents for Transport Assays

See the Supplemental Material.

### Cell Culture for Transwell Transport Assays

Human P-gp–overexpressing MDCKII cells in which the endogenous canine P-gp was knocked out (hMDR1-MDCK^cP-gpKO^) were generously provided by Dr. Per Artursson, Uppsala University. hMDR1-MDCK^cP-gpKO^ cells were cultured in high-glucose Dulbecco’s modified Eagle’s medium that contained 10% FBS, 1% penicillin (10,000 U/ml)/streptomycin (10,000 g/ml), 2 mM Glutamax, and 375 µg/ml Hygromycin B. The human BCRP-overexpressing MDCKII (hABCG2-MDCKII) cells, generously provided by Dr Qingcheng Mao, University of Washington, were cultured in low-glucose Dulbecco’s modified Eagle’s medium that contained 10% FBS, 1% penicillin (10,000 U/ml)/streptomycin (10,000 g/ml), and 500 µg/ml geneticin. Cells were grown at 37°C, 5% CO_2_, and 95% humidity, harvested using trypsin, and subcultured twice a week.

### Transwell Transport Assay

The ER of DEX, BET, DRV (2 µM each), and LPV (0.4 µM [^3^H]LPV + 0.6 µM LPV) was determined in four independent experiments (each conducted in triplicate) in hMDR1-MDCK^cP-gpKO^ cells. ER of DEX and BET (2 µM each) was also determined in four independent experiments (each conducted in triplicate) in hABCG2-MDCKII cells. Quinidine (QND, 3 µM), prazosin (PZS, 3 µM), and Lucifer yellow (LY) were included in the above determinations as markers of robust P-gp, BCRP activity, and integrity of tight junction, respectively. ER was estimated by conducting each experiment in two directions: A→B, in which the donor was the apical (A) compartment (volume = 0.5 ml) and the receiver (B) was the basal compartment (volume = 1 ml), or vice versa (B→A).

Briefly, on day 0, 6 × 10^5^ cells/well were plated on the apical side of the 12-well Transwell polyester insert. Cells were grown in plates for 4 days prior to experiment with the change of medium on day 2. Medium was changed on days 2 and 3. On day 4, cells were washed three times with 37°C transport buffer (10 mM HEPES in HBSS at pH 7.4) and incubated in an orbital shaker at 120 rpm. The donor solution ± tariquidar 5 µM (P-gp inhibitor in hMDR1-MDCK^cP-gpKO^ cells) or ± Ko143 5 µM (BCRP inhibitor in hABCG2-MDCKII cells) was prepared in transport buffer containing the drug and 50 µM paracellular transport marker LY. The receiver solution contained transport buffer ± tariquidar (5 µM) or ± Ko143 (5 µM). Transport assay was initiated by adding the donor solution to the donor compartment and performed at 37°C with 120 rpm shaking. Donor compartments were sampled (10 µl) at time 0 and at the end of the transport experiment. Receiver compartments were sampled (100 µl) at 15, 30, 45, and 60 minutes (DEX, BET); 7, 15, 30, and 45 minutes (DRV); or 60, 120, 180, and 240 minutes (LPV) and replenished with the incubation medium. At the end of each experiment cells were washed three times with ice-cold transport buffer and lysed for drug or marker assay, total protein content (BCA), and proteomic analysis.

### Quantification of Drugs and Markers

[^3^H]LPV was quantified using scintillation counting (PerkinElmer, Waltham, MA). DEX, BET, DRV, QND, and PZS were quantified using liquid chromatography–tandem mass spectrometry (LC-MS/MS) on AB Sciex Triple Quad 6500 (SCIEX, Farmingham, MA) instrument coupled with Waters Acquity ultra performance liquid chromatography (UPLC) system (Waters, Hertfordshire, UK). Briefly, 100 µl of acetonitrile containing 0.5 nM *N*-desmethyl loperamide as internal standard (IS) were added to 50 µl of donor/receiver samples in 96-well plates. Samples were centrifuged at 3220*g*, 4°C, for 15 minutes, and the supernatant was injected into the LC-MS/MS (see Supplemental Tables 1 and 2 for details on LC-MS/MS method and chromatographic conditions). All drug concentrations (diluted where necessary) fell within the linear range of peak area ratios with a signal-to-noise ratio of >5. The permeability of the paracellular marker LY was analyzed on Synergy HTX fluorescence reader (Biotek, Winooski, VT, USA) with excitation/emission wavelength 480/530 nm. The linearity of LC-MS/MS signal (in peak area units) and fluorescence reader signal (in relative fluorescent units) within the quantified work range was confirmed by preliminary experiments (data not shown).

### Determination of In Vitro Efflux Ratios

ER in the absence and presence of P-gp or BCRP inhibitors was determined in the in vitro Transwell assay (eq. 2):

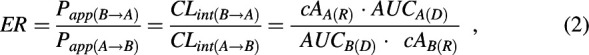

where P_app(B→A)_ and P_app(A→B)_ are apparent permeabilities, and since the surface area is identical in both directions, these are equivalent to CL_int(B→A)_ and CL_int(A→B)_, the apparent intrinsic clearances of a drug in indicated directions; cA_A(R)_ and cA_B(R)_ are cumulative amounts of drug in corresponding receiver compartment, and AUC_A(D)_ and AUC_B(D)_ are AUC of the drug in corresponding donor compartments. cA_A(R)_ and cA_B(R)_ were corrected for the sampled volume at each time point. We used AUC_A(D)_ and AUC_B(D)_ instead of single-donor drug concentration at time 0 because this approach corrects for the depletion of the drug in the donor compartment during the experiment. Only experiments with integral tight junctions [LY apparent permeability (P_app_) < 2·10^−6^ cm/s] were used for further analyses. Likewise, only experiments with ER > 7 for QND or PRZ were included in our analyses. Grouped statistical analysis of ER and P_app_ values was performed by Kruskal-Wallis with Dunn’s multiple comparisons test (*P* < 0.05).

### Prediction of Fetal K_p,uu_ from In Vitro Studies Using the ER-REF Approach

The in vivo K_p,uu_ is related to the clearances mediating the entry and exit of the unbound drug into and from the fetal compartment, respectively, provided fetal elimination of the drug is negligible (see later for justification of this assumption) (eq. 3).

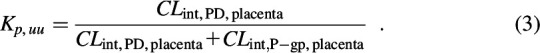


Dividing by CL_int,PD,placenta_ yields the following:

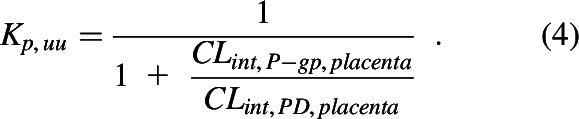


Therefore, the in vivo K_p,uu_ (eq. 4) can be related to the in vitro P-gp–mediated ER as follows:

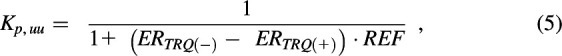

where the ER in the presence and absence of TRQ is the P-gp–mediated ER. To scale this P-gp–mediated ER to that in vivo, the difference in the abundance of P-gp between in vitro (i.e., hMDR1-MDCK^cP-gpKO^ cells) and in vivo should be accounted for. The REF corrects for this difference in abundance. P-gp abundance in cells and in vivo in human placentae was quantified as described below and before (Anoshchenko et al., 2020), respectively.

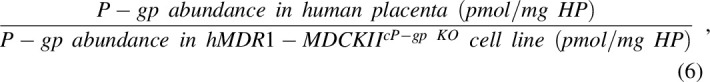

where HP is the total protein in the homogenate of the human placenta or hMDR1-MDCK^cP-gpKO^ cells.

Based on the above equations, when a drug is not a substrate of P-gp and/or BCRP, K_p,uu_ and ER will both equal 1. When a drug is actively effluxed, K_p,uu_ will be <1 and ER >1. The fraction of a drug transported by P-gp (f_t,P-gp_) was then calculated from predicted K_p,uu_ value of each drug (f_t,P-gp_ = 1 – K_p,uu_).

### Quantification of P-gp Abundance in hMDR1-MDCK^cP-gpKO^ Cells and Determination of the REF

After each experiment, cells were lysed on the semipermeable membranes in 1:1 ratio of 2% SDS:EBII buffer for 60 minutes at room temperature; total protein concentration was measured by BCA assay; and approximately 110–160 µg of total protein was reduced, alkylated, and trypsin-digested in duplicate, as described before (Billington et al., 2019; Anoshchenko et al., 2020; Storelli et al., 2020). Ice-cold heavy-labeled IS peptide (NTTGALTTR) was prepared in 80% acetonitrile plus 0.2% formic acid solution and spiked into the trypsin digest (in 1:4 IS:sample ratio) to terminate trypsin digestion. After centrifugation (5000*g*, 4°C), 5 µl of supernatant was injected onto the LC-MS/MS system and analyzed using settings and procedure described before (Anoshchenko et al., 2020). Pooled human placental total membrane sample was used as biologic control and digested with experimental samples. Calibration curve (0.62–40 nM) and quality control samples (0.62, 10, 40 nM) were prepared in 50 mM ammonium bicarbonate buffer, 10 μl of unlabeled peptide standard, and 20 µl of chilled labeled peptide internal standard (both in 80% acetonitrile and 0.2% formic acid solution). P-gp abundance in the homogenate of the term placenta [0.16 ± 0.07 pmol/mg of homogenate protein (Anoshchenko et al., 2020)] was used to estimate the REF value (eq. 6).

### Estimation of Fetal K_p,uu_ Using the Observed In Vivo Data

Fetal in vivo K_p,uu_ of DRV and LPV was estimated as we have previously described for DEX and BET (manuscript in press, Anoshchenko, Milad, and Unadkat). DRV and LPV are usually administered in combination with ritonavir (RTV). The observed DRV and LPV data in nonpregnant and pregnant women (including UV plasma concentrations) are available only for the combination drug dosing regimens, DRV/RTV or LPV/RTV. As an overview (see below for details), we first optimized SimCYP PBPK model of DRV/RTV and LPV/RTV in nonpregnant individuals after oral drug administration of each combination drug regimen. To do so, the model was populated with physicochemical and pharmacokinetic parameters for DRV, LPV, and RTV (Wagner et al., 2017) and verified using the observed drug plasma concentration-time profiles (C-T profiles) in the nonpregnant population (Eron et al., 2004; Boffito et al., 2008; Sekar et al., 2008, 2010). Then, the parameters from nonpregnant population were incorporated into m-f PBPK model and adjusted for pregnancy-induced physiologic changes (e.g., placental and hepatic blood flow, hepatic CYP3A induction, etc.) at the gestational week (average demographic) specified in the observed data sets. Finally, fetal-placental clearance parameters of DRV and LPV were optimized to estimate the in vivo fetal K_p,uu_.

#### Optimization of PBPK Models of DRV and LPV in the Nonpregnant Population.

We first predicted plasma concentration-time (C-T) profiles of DRV administered alone (oral 400 mg twice a day, data not shown), DRV/RTV (oral 600/100 mg twice a day and 800/100 mg every day) and LPV/RTV (oral 400/100 mg twice a day) in the nonpregnant population using SimCYP Simulator version 19 (SimCYP Ltd., A Certara Company, Sheffield, UK). The previously published DRV, LPV, and RTV drug-specific parameters were used (Wagner et al., 2017), except that some of them (t_lag_, k_a_) were optimized (DRV: t_lag_ = 1.3 hours, k_a_ = 0.4 hours^−1^ and LPV: t_lag_ = 1.5 hours) until the predicted steady-state DRV or LPV plasma concentration data adequately described the observed data. The observed DRV or LPV steady-state C-T data (Eron et al., 2004; Boffito et al., 2008; Sekar et al., 2008, 2010) were digitized with WebPlotDigitizer (https://automeris.io/WebPlotDigitizer/). RTV drug-specific parameters included the time-dependent inactivation and induction of CYP3A enzymes in the intestine and the liver.

#### Verification of the m-f PBPK Models of DRV (at GW34 and GW38) and LPV (GW38) in the Pregnant Population.

CYP3A inhibition by RTV in pregnancy was first generated in the SimCYP pregnancy model. Then, the change in bioavailability of DRV or LPV in pregnancy, due to coadministration of RTV (13-fold for DRV and 112-fold for LPV), was incorporated into our m-f PBPK model based on the values determined in SimCYP pregnancy model at the corresponding gestational age. The DRV and LPV steady-state PK parameters obtained in the nonpregnant population were incorporated into our m-f PBPK model built in MATLAB R2020a using our previously published approach (manuscript in press). As per our previous publications, compared with nonpregnant individuals, we assumed maternal hepatic CYP3A activity was induced at term by 2-fold (Hebert et al., 2008; Zhang et al., 2015). For DRV, two sets of maternal C-T profile predictions were generated because of the presence of intensively sampled observed data at GW34 and sparsely sampled data at GW38 (latter, with matching sparsely sampled fetal UV data).

#### Optimization of Fetal-Placental PK Parameters of DRV and LPV at GW38 to Estimate In Vivo Fetal K_p,uu_.

As described before (Zhang and Unadkat, 2017), we estimated the in vivo transplacental passive diffusion clearance (CL_int,PD,placenta_) of DRV and LPV by scaling the in vivo midazolam CL_int,PD,placenta_ by the ratio of the P_app_ of the two drugs in hMDR1-MDCK^cP-gpKO^ cells (1.19 × 10^−5^ and 1.25 × 10^−5^ cm/s, respectively) and that of midazolam (MDZ CL_int,PD,placenta_ = 500 l/h, P_app_ = 4.9 × 10^−5^ cm/s; determined in MDCKII or Caco-2 cells). The resulting DRV and LPV CL_int,PD,placenta_ were 121 and 127 l/h, respectively, values that were much greater than the placental blood flow at term (∼45 l/h). Therefore, DRV and LPV CL_int,PD,placenta_ were considered to be perfusion-limited (45 l/h). Fetal hepatic intrinsic clearance was assumed to be negligible because of low CYP3A7 turnover of CYP3A metabolized drugs and low fetal liver weight (Zhang and Unadkat, 2017) (manuscript in press, Anoshchenko, Milad, and Unadkat). Then, as we have described before (manuscript in press), the in vivo fetal K_p,uu_ value was optimized by adjusting CL_int,P-gp,placenta_ until the predicted unbound UV/MP best described the observed unbound UV/MP [by minimizing the absolute average fold error (AAFE)]. The observed maternal and UV steady-state C-T profiles of DRV were obtained from published literature (Colbers et al., 2015; Stek et al., 2015; Murtagh et al., 2019). These C-T profiles were digitized with WebPlotDigitizer (https://automeris.io/WebPlotDigitizer/). Because the observed C-T profiles of LPV (Cressey et al., 2015; Fauchet et al., 2015) were highly variable, we used the UV and MP C-T profiles predicted by a population pharmacokinetic (PopPK) model that was previously fitted by others to the UV and MP LPV C-T profiles (Cressey et al., 2015; Fauchet et al., 2015). To generate interindividual variability in the plasma C-T profiles, a virtual population of 100 individuals was simulated within m-f PBPK model to generate the mean, 5th and the 95th percentile profiles [90% confidence interval (CI_90%_)].

### Prediction of DRV and LPV Pharmacokinetics in the Pregnant Population at an Earlier Gestational Age (Week 20; GW20)

To illustrate the utility of our model to predict fetal exposure to drugs at earlier gestational age, we predicted the DRV and LPV maternal-fetal profiles at GW20. GW20 was chosen since this is the earliest gestational age at which all the fetal physiologic parameters (e.g., organ volumes, partition coefficients, blood flows) are available. First, the m-f PBPK model was populated with both maternal and fetal physiologic and hepatic CYP3A activity applicable to GW20 using the gestational age–dependent changes in the parameters that we have published previously (Zhang et al., 2015, 2017). Then, CL_int,PD,placenta_ and CL_int,P-gp,placenta_ (at GW20) for both drugs were adjusted for the GW20 placental surface area (Zhang et al., 2017) and total placental P-gp abundance we have previously quantified (Anoshchenko et al., 2020). Finally, GW20 maternal and fetal C-T profiles at steady-state (dose 16) were generated after oral DRV/RTV 600/100 twice daily and oral LPV/RTV 400/100 twice daily.

### Statistical Analyses and Verification of Predictions

Our acceptance criteria for nonpregnant PBPK and m-f PBPK model verifications were to predict pharmacokinetic parameters (C_max_, AUC and clearance) within 0.8- to 1.25-fold of the observed values and AAFE (where available) of <2. Interindividual variability and CI_90%_ (5th and 95th percentiles) for C-T profiles and K_p,uu_ were generated in a virtual population of 100 individuals and included variability only in the maternal system–related parameters. The CI_90%_ of the predicted fetal K_p,uu_ was generated using pooled variance approach, in which the variability in ER and REF (P-gp abundances in vitro cell line and in vivo placental tissue) were included. Verification of the predicted fetal K_p,uu_ (using the ER-REF approach) was deemed successful if the mean predicted fetal K_p,uu_ fell within CI_90%_ of the observed fetal K_p,uu_.

## Results

### ER of DEX, BET, DRV, and LPV in Transwell Assays Using hMDR1-MDCK^cP-gp KO^ or hABCG2-MDCKII Cells.

**Fig. 2.**
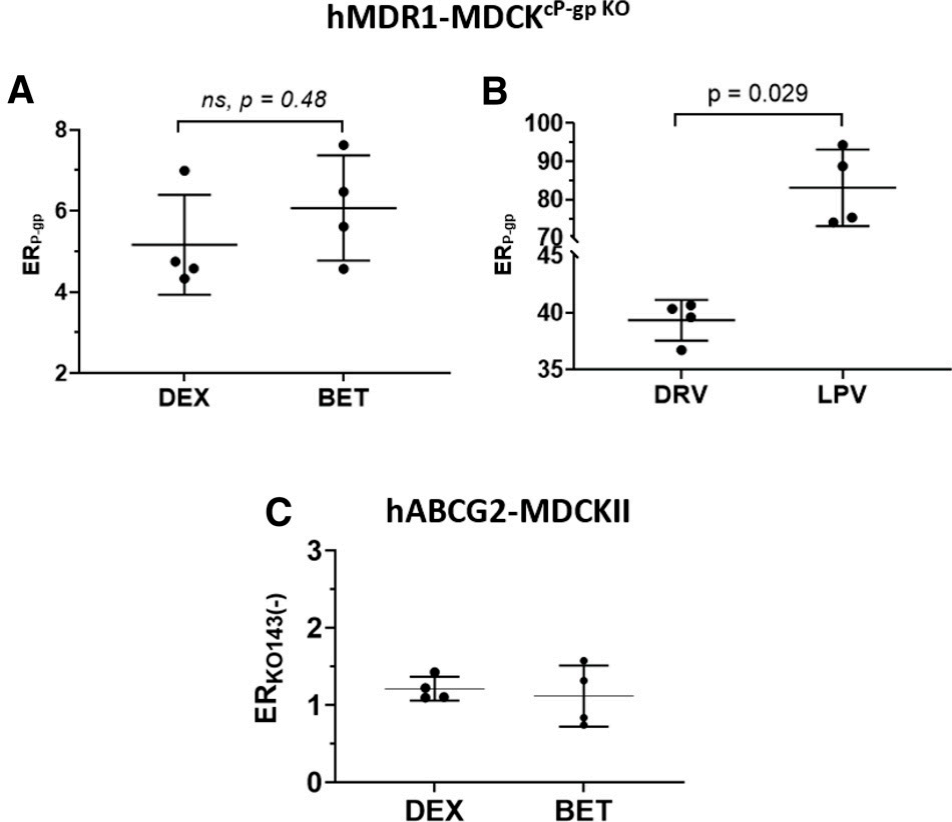
Efflux ratios of test compounds in Transwell assays using monolayer of (A and B) hMDR1-MDCK^cP-gp KO^ or (C) hABCG2-MDCKII. All four drugs were substrates of P-gp in hMDR1-MDCK^cP-gp KO^ cells as evidenced by their P-gp–mediated efflux ratio, ER_P-gp_ [i.e., ER_P-gp_ = ER_TRQ(−)_ − ER_TRQ(+)_]. (A) ER_P-gp_ of DEX (5.1 ± 1.2) and BET (6.1 ± 1.3) were not significantly different, (Kruskal-Wallis test), (B) whereas the ER_P-gp_ of LPV (83.1 ± 10.1) and DRV (39.3 ± 1.8) were significantly different from each other and greater than those of DEX and BET; (C) neither DEX nor BET were substrates of BCRP in hABCG2-MDCKII cells (in the absence of KO143) as evidenced by their efflux ratios of 1.2 ± 0.3 and 1.1 ± 0.1, respectively. Drug concentrations in the donor compartments were 2 µM for DEX, BET, and DRV and 1 µM for LPV. Dots represent individual experiments, each conducted in triplicate; lines represent means and standard deviations. Detailed summary of the efflux ratios of test compounds is provided in Table 1.

DEX, BET, DRV, and LPV were transported by P-gp as evidenced by their P-gp–mediated efflux ratios (ER_P-gp_) in hMDR1-MDCK^cP-gp KO^ cells (Fig. 2; Table 1). In the same experiments, the ER of the positive control QND was 11.1 ± 2.5 (mean ± SD, *n* = 4 experiments, each conducted in triplicate, data not shown). In contrast, DEX and BET were not transported by BCRP. Their ER in hABCG2-MDCKII cells was 1.2 ± 0.3 and 1.1 ± 0.1, respectively (Fig. 2C). In the same experiments, the ER of the BCRP positive control substrate PZS was 7.1 ± 2.5 (mean ± S.D., *n* = 4 experiments, each conducted in triplicate, data not shown). The HIV PIs were not tested in hABCG2-MDCKII cells, as published data indicate that they do not appear to be BCRP substrates (Agarwal et al., 2007; Konig et al., 2010).

**TABLE 1:** ER, REF, and the predicted fetal K_p,uu_ for P-gp Substrates using the ER-REF approach and P-gp overexpressing cells (hMDR1-MDCK^cP-gp KO^) Note that in vivo P-gp abundance used in REF calculations was 0.16 ± 0.07 pmol/mg HP (mean ± S.D.); interexperimental variability in quantification of P-gp protein abundance in the Transwell assays was ∼21%.

Drug	Exp no.	ER_TRQ(−)_	ER_TRQ(+)_	ER_P-gp_	In Vitro P-gp Abundance (pmol/mg protein)	REF	Predicted K_p,uu_		Observed K_p,uu_	Predicted/Observed
				ER_TRQ(−)_ − ER_TRQ(+)_			Value	Mean (CI_90%_)	Mean (CI_90%_)	
DEX	1	5.42	0.85	4.58	1.16	0.14	0.61	0.63 (0.48–0.78)	0.48 (0.30–0.66)	1.31
	2	5.37	1.04	4.33	1.34	0.12	0.66			
	3	8.33	1.35	6.99	1.92	0.08	0.63			
	4	5.65	0.90	4.75	1.20	0.13	0.61			
	Mean ± S.D.	6.2 ± 1.43	1.03 ± 0.22	5.16 ± 1.23	1.41 ± 0.35	0.12 ± 0.02				
BET	1	6.56	0.95	5.61	1.16	0.14	0.56	0.59 (0.42–0.69)	0.5 (0.29–0.71)	1.18
	2	5.64	1.07	4.57	1.34	0.12	0.65			
	3	8.64	1.03	7.62	1.92	0.08	0.62			
	4	7.66	0.92	6.74	1.20	0.13	0.53			
	Mean ± S.D.	7.13 ± 1.31	0.99 ± 0.07	6.13 ± 1.33	1.41 ± 0.35	0.12 ± 0.03				
DRV	1	40.43	0.82	39.61	1.16	0.14	0.15	0.17 (0.10–0.23)	0.16 (0.11–0.22)	1.06
	2	41.83	1.48	40.35	1.34	0.12	0.17			
	3	37.86	1.12	36.74	1.92	0.08	0.25			
	4	41.73	1.06	40.67	1.20	0.13	0.16			
	Mean ± S.D.	40.46 ± 1.85	1.12 ± 0.27	39.34 ± 1.79	1.41 ± 0.35	0.12 ± 0.02				
LPV	1	95.37	1.02	94.35	1.30	0.12	0.08	0.08 (0.07–0.10)	0.11 (0.04–0.19)	0.73
	2	90.07	1.29	88.78	1.20	0.13	0.08			
	3	75.63	1.64	73.99	1.20	0.13	0.09			
	4	76.57	1.30	75.27	0.99	0.16	0.08			
	Mean ± S.D.	84.41 ± 9.84	1.31 ± 0.25	83.1 ± 10.05	1.17 ± 0.13	0.14 ± 0.02				

Observed K_p,uu_, value estimated from in vivo UV/MP ratio at term; Predicted K_p,uu_, value predicted using the ER-REF approach; Exp, experiment.

### Estimates of In Vivo Fetal K_p,uu_ Obtained Using Our m-f PBPK Model.

**Fig. 3.**
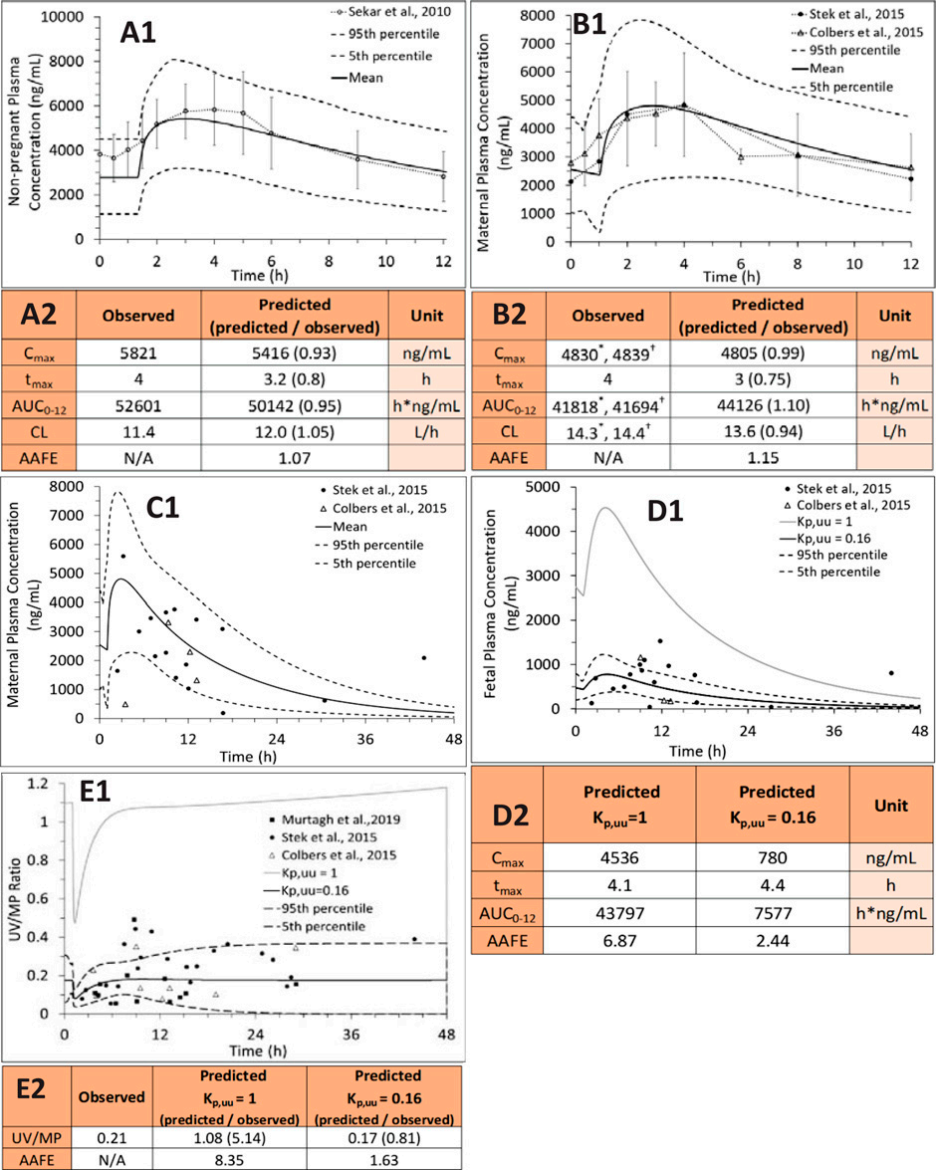
PBPK predictions of DRV steady-state plasma concentrations in (A1) nonpregnant individuals, (B1) pregnant women at GW34 (intensively sampled), (C1) pregnant women at GW38 (sparsely sampled) and their (D1) fetuses at GW38 (sparsely sampled), and (E1) UV/(MP ratio at GW38 with and without incorporation of placental P-gp efflux. Subjects were administered DRV/RTV 600/100 mg oral twice daily. (A1) SimCYP or (B1 and C1) m-f PBPK predicted mean concentration-time profile (solid line) and CI_90%_ (dashed lines) are overlaid on the observed data [intensively sampled (A1) circles: mean ± S.D., *n* = 8; (B1) circles: mean ± S.D., *n* = 32, triangles: mean ± S.D., *n* = 6; or (C1) sparsely sampled]. (D1 and D2) The observed fetal UV concentration-time data were better predicted by our m-f PBPK model in the presence of P-gp efflux clearance (K_p,uu_ = 0.16, black solid line; dashed lines, 5th and 95th percentile profiles) vs. in the absence of P-gp efflux clearance (i.e., passive diffusion only resulting in K_p,uu_ = 1, gray solid line). (E1) The m-f PBPK model better predicted UV/MP ratios in the presence of P-gp efflux clearance (K_p,uu_ = 0.16) vs. in the absence of P-gp efflux clearance (K_p,uu_ = 1). The observed UV/MP ratios are combined from two dosing regimens of DRV/RTV: 600/100 twice daily and 800/100 every day to increase the confidence in our model verification as these ratios are independent of dosing regimen. (A2, B2, D2 and E2) The predicted pharmacokinetic parameters in (A2) and (B2) met our a priori defined acceptance criteria (within 0.8- to 1.25-fold of the observed data). The observed PK parameters were estimated from Stek et al. (2015)* or Colbers et al. (2015)^†^.

**Fig. 4.**
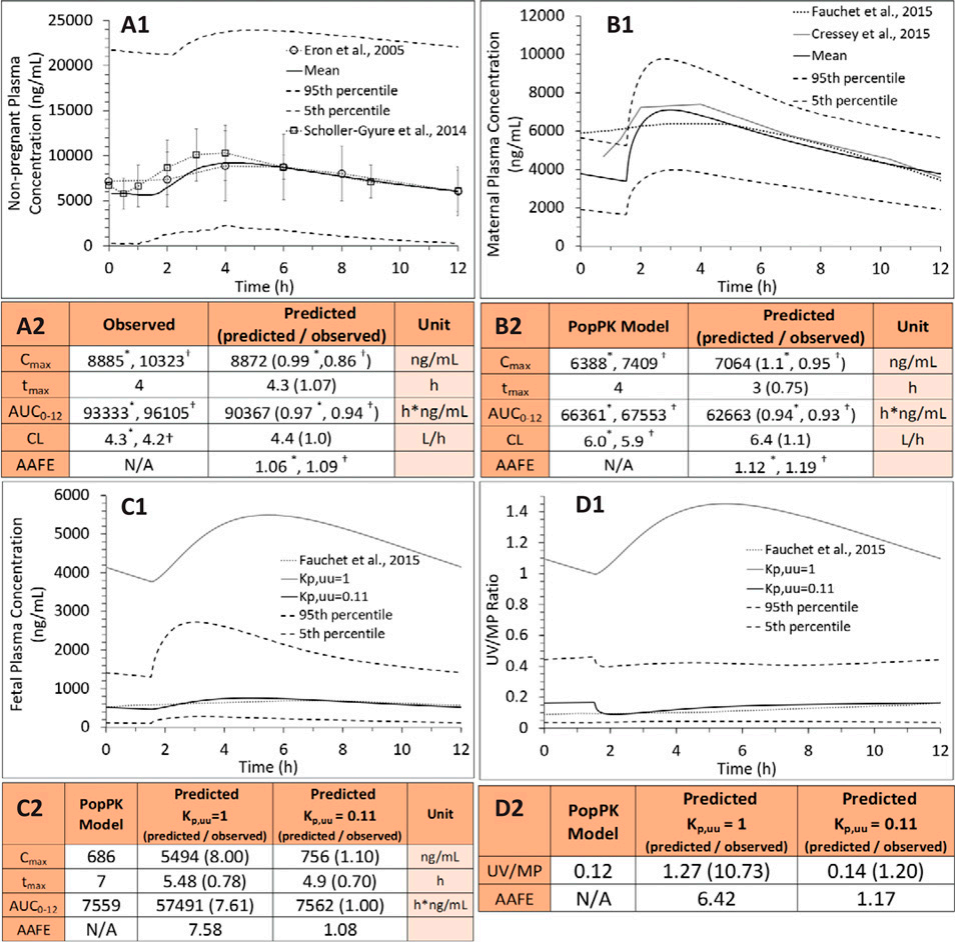
PBPK predictions of LPV steady-state plasma concentrations in (A1) nonpregnant individuals, (B1) pregnant women, and (C1) their fetuses at GW38 and (D1) UV/ MP ratio with and without incorporation of placental P-gp efflux. Subjects were administered LPV/RTV 400/100 mg oral twice daily. (A1 and B1) SimCYP or m-f PBPK predicted mean concentration-time profile (solid line) and CI_90%_ (dashed lines) are overlaid on the observed data [(A1) circles: mean ± S.D., *n* = 19; squares: mean ± S.D., *n* = 16) or (B1) two published PopPK profiles, respectively (gray solid line)]. (C1 and C2) The “observed” (i.e., PopPK predicted) fetal UV concentration-time profile (dotted line) was better predicted by our m-f PBPK model in the presence of P-gp efflux clearance (K_p,uu_ = 0.11, black solid line; dashed lines, 5th and 95th percentile profiles) vs. in the absence of P-gp efflux clearance (i.e., passive diffusion only resulting in K_p,uu_ = 1, gray solid line). (D1) The m-f PBPK model better predicted the “observed” (i.e., PopPK predicted) UV/MP ratios in the presence of P-gp efflux clearance (K_p,uu_ = 0.11) vs. in the absence of P-gp efflux clearance (K_p,uu_ = 1). (A2, B2, C2, and D2) The predicted pharmacokinetic parameters met our a priori defined acceptance criteria (within 0.8–1.25 of the observed or PopPK predicted). The published PopPK parameters were estimated from (A2) Eron et al. (2004)* and Scholler-Gyure et al. (2013)†, or (B2) Fauchet et al. (2015)* or Cressey et al. (2015)^†^.

To estimate the in vivo fetal K_p,uu_ (to verify our ER-REF predictions), we first successfully predicted C-T profiles and pharmacokinetic parameters of LPV and DRV in the nonpregnant population after oral DRV/RTV 600/100 twice daily (Fig. 3, A1 and A2), DRV/RTV oral 800/100 every day (Supplemental Fig. 3, A1 and A2), or LPV/RTV oral 400/100 twice daily (Fig. 4, A1 and A2). Then, using our m-f PBPK model (which incorporates pregnancy-induced changes in pharmacokinetic and physiologic parameters at gestational week (average demographic) specified in observed data sets, we predicted the C-T profiles of LPV (GW38: Fig. 4B1) or DRV (GW34: Fig. 3B1; GW38 Fig. 3C1) in pregnant women who were administered the above dosing regimens. The predicted C-T profiles in pregnant women were successfully verified, as evidenced by comparing the predicted and observed data (Figs. 3B1 and 4B1: predicted CI_90%_ captured observed/PopPK predicted data; Fig. 3C1: AAFE = 1.93 and Supplemental Fig. 3C1: AAFE = 1.72) and the predicted pharmacokinetic parameters falling within 0.8- and 1.25-fold of the observed data (our predefined acceptance criteria) (Fig. 3B2, Supplemental Fig. 3B2, and Fig. 4B2, respectively).

Once the maternal C-T profiles were verified, we optimized the in vivo placental P-gp–mediated efflux clearance (CL_int,P-gp,placenta_) for DRV and LPV using our m-f PBPK model and published UV/MP data at term (Figs. 3 and 4). For DRV, in vivo placental efflux clearance (CL_int,P-gp,placenta_ = 612 l/h), yielding K_p,uu_ = 0.16, resulted in the best prediction of UV/MP ratio (AAFE = 1.63) compared with when no CL_int,P-gp,placenta_ was invoked (AAFE = 8.35, K_p,uu_ = 1) (Fig. 3, E1 and E2). For LPV, in vivo placental efflux clearance (CL_int,P-gp,placenta_ = 1029 l/h) yielding K_p,uu_ = 0.11 resulted in the best prediction of UV/MP ratio (AAFE = 1.17) compared with when no CL_int,P-gp,placenta_ was invoked (AAFE = 6.42, K_p,uu_ = 1) (Fig. 4, D1 and D2). DEX and BET in vivo K_p,uu_ were similarly estimated (0.48 and 0.5, respectively) and obtained from our submitted publication.

### Prediction and Verification of Fetal K_p,uu_ Using the ER-REF Approach.

**Fig. 5.**
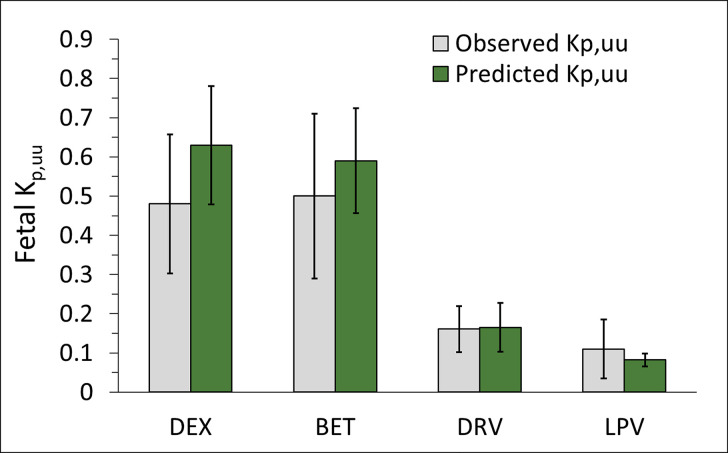
Successful prediction of fetal K_p,uu_ by the REF-ER approach when compared with the in vivo K_p,uu_ estimated by m-f PBPK modeling and simulation of the observed data. The mean ER-REF predicted K_p,uu_ values of DEX, BET, DRV, and LPV (green bars, error bars are CI_90%_) fell within CI_90%_ (error bars) of the mean observed values (gray bar), demonstrating the success of the ER-REF approach.

After the in vitro ER of DEX, BET, DRV, and LPV were scaled using the ER-REF approach (eqs. 5 and 6), the predicted in vivo fetal K_p,uu_ (mean and CI_90%_) obtained were 0.63 (0.48–0.78), 0.59 (0.42–0.69), 0.17 (0.1–0.23), and 0.08 (0.07–0.1), respectively (Fig. 5; Table 1). The mean ER-REF predicted values fell within CI_90%_ of estimated from in vivo values for DEX (0.3–0.66), BET (0.29–0.71), DRV (0.11–0.22), and LPV (0.04–0.19), demonstrating success of the ER-REF approach (Fig. 5; Table 1). These mean ER-REF predicted K_p,uu_ resulted in UV/MP ratio profiles that predicted the observed values well described (DRV, LPV; Supplemental Fig. 4, A and B) or modestly overpredicted the observed values (BET, DEX; Supplemental Fig. 4, C and D). These ER-REF predicted K_p,uu_ values yielded mean in vivo fraction of drug transported by placental P-gp (f_t,P-gp_ = 1 – K_p,uu_) of 0.37, 0.41, 0.84, and 0.92 for DEX, BET, DRV, and LPV, respectively.

### Prediction of DRV/RTV and LPV/RTV K_p,uu_ at an Earlier Gestational Age (GW20).

**Fig. 6.**
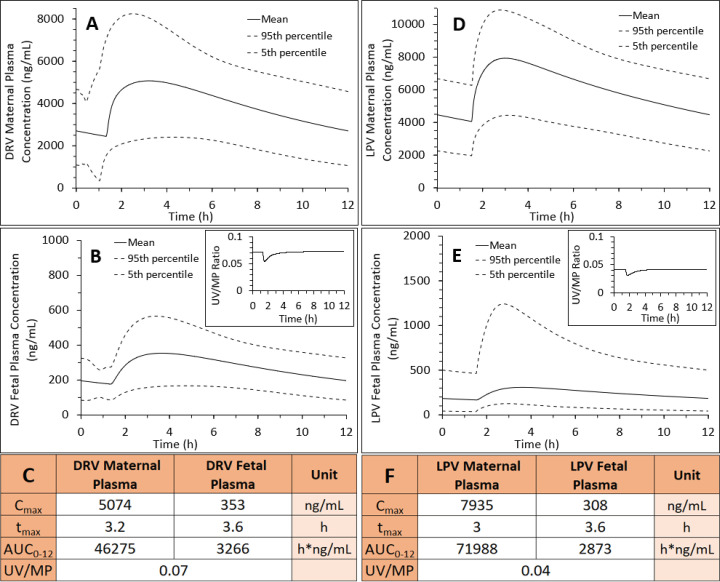
M-f PBPK model predictions of DRV or LPV steady-state plasma drug concentrations at GW20 after administration of (A–C) 600/100 mg oral DRV/RTV twice daily or (D–F) 400/100 mg oral LPV/RTV twice daily. (B and C) Fetal plasma DRV C_max_ and AUC_0-12_ at GW20 were 45% and 43% of that at GW38 (Fig. 3, D1 and D2), whereas maternal plasma DRV C_max_ and AUC_0-12_ at GW20 (A and C) were approximately the same as that at GW38 (Fig. 3, B1 and B2), indicating that both P-gp efflux and passive diffusion clearance affect fetal rather than maternal DRV exposure. These values yielded DRV K_p,uu_ of 0.11 at GW20 versus K_p,uu_ of 0.16 at GW38. (B, inset, and C) DRV UV/MP ratio at GW20 was 41% of that at GW38 (Fig. 3, E1 and E2). (E and F) Fetal plasma LPV C_max_ and AUC_0-12_ at GW20 were 41% and 38% of that at GW38 (Fig. 4, C1 and C2), whereas maternal plasma LPV C_max_ and AUC_0-12_ at GW20 (D and F) were only modestly (1.12- and 1.15-fold, respectively) higher than at GW38 (Fig. 4, B1 and B2). These values yielded LPV K_p,uu_ = 0.07 at GW20 vs. K_p,uu_ of 0.11 at GW38. (E, inset, and F) LPV UV/MP ratio at GW20 was 29% of that at GW38 (Fig. 4, D1 and D2).

At GW20, CL_int,PD,placenta_ values for DRV and LPV were 47 and 49.5 l/h, respectively (calculated from term CL_int,PD,placenta_ values by adjusting for the change in placental surface area between two gestational ages). These values exceeded placental blood flow at this gestational age (27.5 l/h), yielding perfusion-limited CL_int,PD,placenta_. CL_int,P-gp,placenta_ at GW20, adjusted for decrease in total placental P-gp abundance at this gestational age (Anoshchenko et al., 2020), resulted in values 40% lower than the corresponding values at GW38 (367 and 617 l/h for DRV and LPV, respectively). After gestational age adjustment of other maternal-fetal physiologic and pharmacokinetic parameters, the m-f PBPK model predicted fetal DRV and LPV UV plasma AUCs were, respectively, 43% and 38% of that at GW38. In contrast, the corresponding maternal plasma AUC of DRV was unchanged, whereas that of LPV was modestly, 1.15-fold, higher at GW20 than at GW38 (Fig. 6). These changes predicted DRV and LPV fetal K_p,uu_ values at GW20 of 0.11 and 0.07, respectively (69% and 64% of that at GW38).

## Discussion

Using our m-f PBPK model, we have successfully predicted and verified fetal exposure to drugs that passively cross the placenta (Zhang and Unadkat, 2017). However, pregnant women often take drugs that are effluxed by placental transporters. We have previously shown that the REF approach can successfully predict transporter-based clearance and tissues concentration of drugs (Ishida et al., 2018; Kumar et al., 2018, 2021; Sachar et al., 2020; Storelli et al., 2021). Similarly, here we determined whether our ER-REF approach, combined with our m-f PBPK model, could predict fetal exposure to drugs that are transported by placental transporters. We chose to test this hypothesis using the placental P-gp transporter as our model transporter because, of all the transporters expressed in the placenta, it is arguably the most important in modulating fetal drug distribution. This is because it is highly abundant in the human placentae (Mathias et al., 2005; Joshi et al., 2016; Anoshchenko et al., 2020) and is capable of transporting wide variety of marketed drugs (Schinkel and Jonker, 2003). Indeed, many drugs (e.g., antibiotics, cardiac drugs, antiemetics, HIV drugs) taken by pregnant women are effluxed by placental P-gp. Here, using the ER-REF approach, combined with our m-f PBPK model, we present the first successful prediction of fetal K_p,uu_, at term, for drugs that are transported by the human placentae. Moreover, our predicted fetal K_p,uu_ were verified by data observed at term. Although we would have preferred to conduct verification of our prediction at several gestational ages, such verification is not possible as a result of unavailability of UV and MP data at gestational ages other than term.

Our ER-REF approach deliberately incorporated several elements to enhance our success in K_p,uu_ predictions. First, we used transfected MDCK cell line that had the endogenous canine P-gp knocked out. Therefore, our measured ER and predicted fetal K_p,uu_ were not confounded by endogenous canine P-gp activity. Second, we measured P-gp abundance in hMDR1-MDCK^cP-gpKO^ cells in each independent transport experiment, and hence, our REF was not confounded by differences in in vitro transporter abundance between cell passage numbers (Table 1). Third, the quantification of P-gp abundance in vitro was performed using the same method as for in vivo placental tissue (Anoshchenko et al., 2020), within the same laboratory, hence minimizing bias (due to interlaboratory variability in proteomics quantification) in determining REF. Fourth, we chose to study drugs that were selective for a given transporter—namely, P-glycoprotein. Thus, the presence of other transporters in the placenta (e.g., BCRP) did not confound the observed or predicted in vivo fetal K_p,uu_. Indeed, we showed that the ACS were not substrates of BCRP (ER < 2 in hABCG2-MDCKII cells; Fig. 2C). And, literature data suggest that the PIs, DRV and LPV, are also unlikely substrates of BCRP (Agarwal et al., 2007; Konig et al., 2010). Fifth, none of the drugs are likely to be significantly metabolized in placenta, which would also confound interpretation of the in vivo K_p,uu_. All four drugs are primarily metabolized by CYP3A, the enzyme with relatively low placental abundance and activity (Myllynen et al., 2009; Pasanen, 1999; Myllynen et al., 2007). Besides CYP3A, DEX and BET can also be metabolized by 11 *β*-hydroxysteroid dehydrogenase-2 enzyme present in placenta, although the rate and extent of such metabolism relative to CL_int,PD,placenta_ and CL_int,P-gp,placenta_ is low (e.g., ∼10%–15% of DEX/BET metabolized over 6 hours in vitro in placental microsomes)(Blanford and Murphy, 1977; Murphy et al., 2007). Sixth, we confirmed that the ER of the ACS drugs in our Transwell assays was independent of concentration (over the range 2–250 µM). Because of low solubility of DRV and LPV (16 and 3 µM, respectively; DrugBank database), a similar study over a wide range of concentrations was not feasible. Therefore, for our Transwell assays we selected the lowest concentration of all four drugs that was quantifiable by our analytical method (2 µM for DEX/BET/DRV and 1 µM for LPV). Although RTV has been reported to be a P-gp inhibitor, based on the reported in vivo plasma concentration of the drug at the doses administered together with DRV or LPV, it is highly unlikely to inhibit placental P-gp in vivo. The highest reported maternal plasma RTV unbound C_max_ is 13 nM (Stek et al., 2015) (at 100 mg, twice daily), much lower than the lowest reported RTV IC_50_ for P-gp [240 nM (Vermeer et al., 2016)]. Additionally, in vivo data (Gimenez et al., 2004) also support that low-dose RTV is unlikely to inhibit brain P-gp in human (Tayrouz et al., 2001) or mice (Huisman et al., 2001; Gimenez et al., 2004). Therefore, in determining DRV or LPV ER in hMDR1-MDCK^cP-gp KO^ cells, RTV was not added to the donor compartment. Seventh, interestingly, although the in vivo K_p,uu_ of the PIs was estimated from data obtained when they were coadministered with RTV (a potent intestinal CYP3A inhibitor), incorporating 2-fold induction of hepatic CYP3A4 in pregnancy (Hebert et al., 2008) into the m-f PBPK model, did not result in a proportional 2-fold increase in PI’s maternal clearance. Instead, the increase was rather modest: 1.1-fold for DRV and 1.5-fold for LPV. The reason for this observation is likely due to inhibition of hepatic (and intestinal) CYP3A enzymes by RTV (Kirby et al., 2011). And incorporation of such inhibition in our m-f PBPK recapitulated the observed increase in maternal clearance of 1.2-fold and 1.4-fold, respectively (Figs. 2, B–D and 3, B–D). Finally, our prediction of K_p,uu_ was based on UV/MP values, values that are obtained from multiple maternal-fetal dyads, rather than on UV values alone. This is because significant interindividual variability in maternal plasma concentration can result in significant interindividual variability in UV C-T profile. However, this variability is considerably mitigated when UV/MP values are used.

Our in vitro findings confirmed previous data (Ueda et al., 1992; Crowe and Tan, 2012; Prasad and Unadkat, 2015) that all four drugs are moderate to excellent P-gp substrates [defined by the Food and Drug Administration as efflux ratios of >2 in P-gp–overexpressing cell lines (US Food and Drug Administration, 2017)] (Fig. 2; Table 1). As expected, because DEX and BET are epimers, their efflux ratios in the P-gp–overexpressing cell line and the corresponding predicted fetal K_p,uu_ were not significantly different (Fig. 2A; Table 1), consistent with their similar in vivo K_p,uu_ (manuscript in press, Anoshchenko, Milad, and Unadkat). Based on these data, the estimated in vivo f_t,P-gp_ for DEX and BET were 0.52 and 0.50, respectively. LPV showed higher ER (hence, lower ER-REF predicted K_p,uu_, or alternatively, higher f_t,P-gp_) than DRV (Fig. 2B; Table 1). Hence, our in vitro predictions (in agreement with DRV and LPV in vivo K_p,uu_ observations; Figs. 3, E–F and 4, E–F, respectively) indicate lower fetal LPV exposure at term compared with DRV. Also, placental P-gp drug efflux resulted in decreased fetal drug exposure to all four drugs (K_p,uu_ < 1; Fig. 5) when compared with their corresponding fetal exposure (K_p,uu_ = 1) if only passive placental diffusion of the drug was assumed.

The mean ER-REF predicted K_p,uu_ values were in good to excellent agreement with the estimated in vivo K_p,uu_ values, demonstrating success of the ER-REF approach (Fig. 5; Table 1). For DEX and BET, the observed in vivo K_p,uu_ was modestly overpredicted by the ER-REF approach. This success enhances confidence in using our ER-REF approach to predict fetal exposure to drugs at earlier gestational ages. This is important because many drugs (e.g., DRV, LPV) are administered to pregnant women earlier in gestation and/or throughout pregnancy. Indeed, our m-f PBPK model predicted lower fetal exposure to DRV or LPV at GW20 versus term (Fig. 6). This finding is a result of an interplay between two clearance processes defining transplacental passage of the drugs (eq. 4). Alternatively stated, it is the ratio of CL_int,P-gp,placenta_ and CL_int,PD,placenta_ that determines K_p,uu_ of drugs. Although P-gp abundance per gram of placenta is higher at GW20 versus term, because the placenta size is smaller at GW20 versus term, the abundance of P-gp in the whole placenta is also lower at GW20 versus term. Both the size and total placental P-gp abundance at GW20 versus term resulted in a greater decrease in CL_int,PD,placenta_ of the drugs (↓80%, due to lower placental surface area) than in the decrease in CL_int,P-gp,placenta_ (↓40%, due to lower total P-gp abundance), resulting in lower predicted in vivo K_p,uu_ of the drugs at GW20 versus term. Unfortunately, the predicted fetal drug exposure at GW20 cannot be verified because of the lack of observed UV data. Nevertheless, these predictions demonstrate the ability of our m-f PBPK model to predict fetal exposure to drugs at earlier gestational ages.

There are several limitations to our study. First, verification of LPV K_p,uu_ was challenging because of the large variability in the maternal-fetal data. Hence, we resorted to the use of previously published PopPK model predictions. When data for additional drugs appropriate for PBPK modeling are available (criteria for such data sets were described before in the manuscript in press, Anoshchenko, Milad, and Unadkat), we will be able to verify our model with greater confidence and for additional P-gp substrates. Second, we modestly overpredicted DEX UV/MP ratio profile based on the ER-REF predicted K_p,uu_ value (Supplemental Fig. 4D). This overprediction may be due to lack of observed UV/MP values over a duration necessary to accurately estimate its K_p,uu,_ involvement of efflux transporters other than P-gp or BCRP or metabolism in the placenta. Third, we could not predict fetal exposure to drugs at <GW20, as fetal physiologic parameters are not reliably available at <GW20 (Zhang et al., 2017; Abduljalil et al., 2019). Additionally, the lack of established maternal-placental blood circulation before GW13 (Chang et al., 2018) (restricting overall drug access to the fetus), limits out model application to the second and third trimester of pregnancy.

Despite the high prevalence of drug use in pregnancy [∼80% of pregnant women using at least one drug (Scaffidi et al., 2017)], 90% of drugs on the market still lack guidance on their administration in this population, leaving both mother and her fetus “drug orphans.” Although we have some understanding of maternal drug exposure (and changes therein) during pregnancy (Anderson, 2005; Hebert et al., 2008; Abduljalil et al., 2012, 2020), this is not the case for fetal drug exposure, which is related to fetal drug efficacy and toxicity. This study is the first to address this significant gap in health care knowledge, that is development of a method to successfully predict fetal exposure to drugs irrespective of whether they are transported or not. Since UV/MP data at term are not readily available for all drugs prescribed to pregnant women, and since such studies are logistically and ethically challenging to conduct, our approach provides a means to predict fetal exposure to drugs, irrespective of whether they diffuse across the placenta or are transported. Moreover, together with placental transporter abundance that we have previously quantified (Anoshchenko et al., 2020), this ER-REF approach can be used to predict fetal exposure to placental transported drugs at gestational ages other than term (as shown here for GW20). Our ER-REF scaling approach can easily be adapted to substrates of multiple placental transporters (e.g., P-gp and/or BCRP), as has been shown before for transporter-mediated uptake and distribution of drugs to various organs (Trapa et al., 2016, 2019; Ishida et al., 2018; Kumar et al., 2018, 2021; Sachar et al., 2020; Storelli et al., 2021). In conclusion, our study provides a tool to prospectively predict the fetal exposure to drugs at various gestational ages to help assess potential fetal benefits and risks associated with maternal drug administration.

## Abbreviations

A: apical compartment
AAFE: absolute average fold error
ACS: antenatal corticosteroid
AUC: area under the curve
B: basal compartment
BCRP: breast cancer resistance protein
BET: betamethasone
CI_90%_: 90% confidence interval spanning between 5th and 95th percentiles
CL_int,PD,placenta_: intrinsic placental passive diffusion clearance
CL_int,P-gp,placenta_: in vivo P-gp–mediated efflux clearance from the placenta
C-T profile: drug plasma concentration-time profile
DEX: dexamethasone
DRV: darunavir
ER: efflux ratio
ER_P-gp_: P-gp–mediated efflux ratio
ER-REF: efflux ratio–relative expression factor
f_t,P-gp_: fraction of a drug transported by P-glycoprotein
GW: gestational week
hABCG2-MDCKII: Madin-Darby canine kidney cells II with overexpressed human ABCG2 [BCRP]
HIV: Human immunodefficiency virus
hMDR1-MDCK^cP-gp KO^: Madin-Darby canine kidney II cells with overexpressed human multidrug resistance protein 1 [P-gp] and knocked out canine P-gp
IS: internal standard
k_a_: absorption rate constant
K_p_: partition coefficient
K_p,uu_: unbound partition coefficient
LC-MS/MS: liquid chromatography–tandem mass spectrometry
LPV: lopinavir
LY: Lucifer yellow
MDCK: Madin-Darby canine kidney
m-f PBPK model: maternal-fetal physiologically based pharmacokinetic model
MP: maternal plasma
P_app_: apparent permeability
PI: HIV protease inhibitor
PK: pharmacokinetic
PopPK: population pharmacokinetic
PZS: prazosin
QND: quinidine
REF: relative expression factor
RTV: ritonavir
SYT: syncytiotrophoblast
t_lag_: lag time
TRQ: tariquidar
UV: umbilical vein
